# Interplay between γδT-Cell Metabolism and Tumour Microenvironment Offers Opportunities for Therapeutic Intervention

**DOI:** 10.20900/immunometab20210026

**Published:** 2021-07-30

**Authors:** Marta Barisa, Daniel Fowler, Jonathan Fisher

**Affiliations:** Zayed Centre for Research, Great Ormond Street Hospital, University College London Great Ormond Street Institute of Child Health, London WC1N 1DZ, UK

**Keywords:** γδT cells, gamma delta T cells, oncoimmunology, immunotherapy, solid tumours, immunometabolism

## Abstract

Solid tumour targeting using adoptive cell therapy has failed to reproduce the spectacular clinical successes seen with chimeric antigen receptor T cell therapies and B cell malignancies. Low in glucose, oxygen, pH and populated with suppressive cells, the solid tumour microenvironment (TME) remains a formidable obstacle to successful immune targeting. The use of atypical, tissue-tropic lymphocytes, such as γδT cells, may offer enhanced tumour trafficking over canonical αβT cells. Nonetheless, γδT cells too interact with the TME. The consequences of this interaction are poorly understood and of high translational relevance. Lopes and colleagues show that, in a murine context, low glucose environments preferentially retained pro-tumorigenic IL-17-producing γδT cells. Anti-tumorigenic IFN-γ-producing γδT cells, meanwhile, required high ambient glucose to survive and exert effector function. Unexpectedly, this metabolic imprinting was evident in the murine thymus, suggesting that the ontological separation of these functional subsets occurs early in their development. Elucidation of this relationship between TME glucose levels and γδT cell functionality in a human context is likely to carry significant implications for the development of γδT cell-based oncoimmunotherapeutics.

The proof of concept that adequately engineered T cells have the capacity to destroy advanced cancer and induce long-term remission was provided by the clinical successes of chimeric antigen receptor (CAR)-T cell therapies targeting cell surface antigens in B cell leukaemias and lymphomas [1,2]. Despite significant initial optimism, reproducing sustained T cell-mediated clinical benefits in a solid tumour context has proven problematic. A range of obstacles have since been identified as hampering solid tumour targeting, including a composite of physical and chemical barriers to access of T cells, the presence of suppressive immune and tumour cells, variable target antigen expression and low mutational burden [2–4].

Cellular immunotherapy design has canonically focused on classical alpha/beta (αβ)T cells, characterized by their expression of an alpha/beta T cell receptor (TCR) heterodimer. High numbers of cytotoxic αβT cells reside in peripheral circulation and are amenable to high efficiency gene editing, providing some of the rationale for their success as therapeutic tools targeting haematological malignancies. Their efficacy against solid tumours has proven less reliable. Promising novel avenues for solid tumour targeting have sought to explore lymphocyte populations with more favourable tissue-tropic homing profiles, including T cells that express a gamma/delta TCR heterodimer, gamma/delta (γδ)T cells. Remarkably, a meta-analysis of expression signatures from ~18,000 human tumours with overall survival outcomes across 39 malignancies identified γδT cells as the single most positive lymphocytic prognostic factor for patient outcomes across all cancers investigated [5].

Nonetheless, while exhibiting a suitable safety profile—unmodified, ex vivo-expanded γδT cell adoptive transfusions have shown little sustained efficacy against established solid malignancy in clinical trials of both autologous or allogeneic origin, leaving much room for therapeutic improvement [6,7]. Poor cell product persistence, proliferation, survival inside and suppression by the tumour microenvironment (TME) are all likely contributors to the apparent subpar fitness of the cells. While mounting evidence elucidates the various avenues through which αβT cell metabolism acts as a determinant of cell fitness [8,9], the link between γδT cell metabolism and functionality remains almost entirely unknown. A recent study by Lopes et al. sheds light on this understudied and translationally relevant area.

The authors aimed to elucidate the metabolic cues that regulate the balance between different γδT cell subsets in the TME, and how these impact resulting cell effector phenotype and consequent tumour resolution. The study focused on the predominant and ontologically separate effector duality of murine γδT cells: the largely anti-tumourigenic IFN-γ producers (γδ^IFN^) and the largely pro-tumourigenic IL-17 producers (γδ^17^) [10]. Direct study of peripheral, thymic and TME-resident murine γδT cells as well as glucose deprived or supplemented culture model systems were employed, documenting the relative enrichment of γδ^IFN^ or γδ^17^ cells and their metabolic profile, as measured by dependence on and employment of oxidative phosphorylation (OXPHOS), glycolysis or fatty acid oxidation (FAO). γδ^IFN^ presented with an almost exclusively glycolytic phenotype, while γδ^17^ were highly dependent on mitochondrial and lipid oxidative metabolism (Figure 1). In contrast to canonical αβT cells, which acquire distinct metabolic profiles in the periphery following activation [9], γδT cell metabolic (and functional) dichotomy appeared to be already established during thymic development, and subsequently maintained in the periphery and the TME.

Most solid tumours are associated with high glycolytic activity and compete directly with infiltrating lymphocytes for glucose, with documented dramatic consequences on αβT cell metabolism and functionality [11]. Lopes et al. establish one of the first lines of evidence that a similar relationship exists for γδT cells: a glucose-low, lipid-rich TME led to a striking and selective enrichment of γδ^17^, but not γδ^IFN^. Glucose-high environments, meanwhile, lead to preferential retention and expansion of the γδ^IFN^ subset. This presents both a substantial and serious obstacle for γδT cell solid tumour targeting as well as a potential therapeutic opportunity. In that vein, the authors designed a basic intervention that consisted of γδT cell ex vivo expansion in glucose-enriched media as opposed to standard culture media. Consistent with their earlier findings, in vitro glucose supplementation led to a greater accumulation of γδ^IFN^ cells and subsequent enhanced in vivo antitumor functionality of the cell product. This carries potentially significant implications for the successful clinical translation of γδT cell therapies for solid tumour indications.

Lopes and colleagues provide valuable insight into the as yet poorly understood relationship between metabolism and murine γδT cell functionality, offering strategies for enhancing tumour targeting. While of substantial interest, it must be noted that murine and human γδT cell biology diverges in a range of well-documented areas [12]. Ultimate relevance of these findings to human γδT cells and, therefore, translation remains to be established. Likewise, further work is necessary to establish the durability of glucose-mediated enhancement of γδT cell anti-tumour functionality. Mechanistic insight into the relationship between metabolism and phenotype is emerging as a valuable asset in cell therapy design. Unravelling this complex relationship is likely to be a dynamic and productive area of translational γδT cell study for years to come.

## Figures and Tables

**Figure 1:**
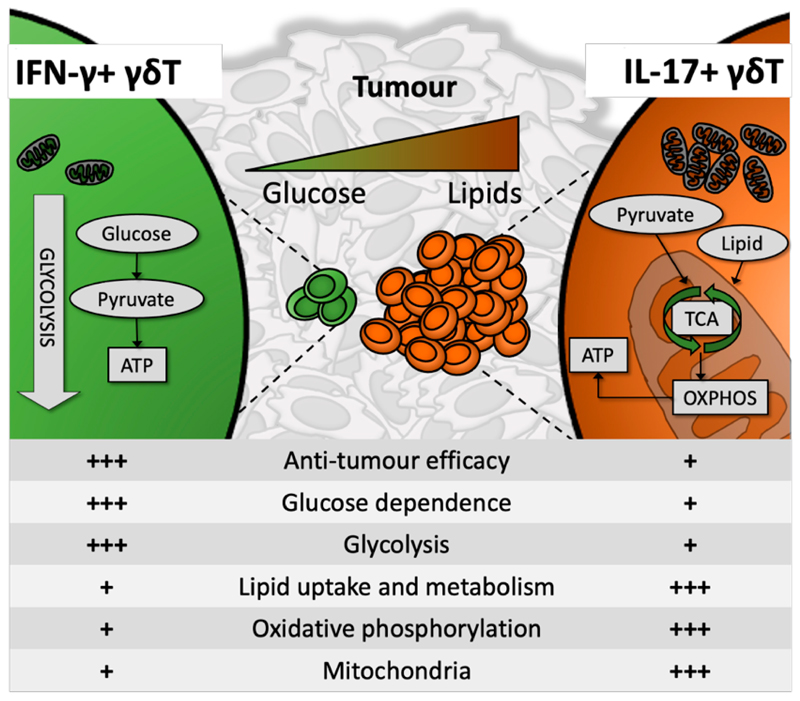
Anti-tumorigenic IFN-γ-producing γδT cells (γδ^IFN^) contained few mitochondria and were highly glycolysis-dependent, preferentially persisting and thriving in a glucose-high environment. In contrast, IL-17-producing γδT cells presented with high mitochondrial mass, were preferentially reliant on oxidative metabolism and utilized lipids for energy synthesis. This has potentially significant implications for antitumour γδ^IFN^ efficacy inside the lipid-rich, glucose-poor solid tumour microenvironment.
